# Capillary stalling: A common microvascular pathway to stroke susceptibility and poor recovery

**DOI:** 10.1016/j.nbd.2026.107460

**Published:** 2026-05-27

**Authors:** Mikaela A. Barbour, Kate Karelina, Zachary M. Weil

**Affiliations:** Department of Neuroscience and Rockefeller Neuroscience Institute, West Virginia University, 108 Biomedical Road, 313 BMRC, Morgantown, WV 26506, USA

**Keywords:** Stroke, Inflammation, Coagulation, Cerebrovascular disease, Microvasculature

## Abstract

Capillary stalling, the transient interruption of blood flow within individual capillaries, is a potential indicator of cerebral microvascular dysfunction and may contribute to stroke susceptibility and recovery. Stroke remains a leading cause of death and disability worldwide, yet many patients do not qualify for available interventions, underscoring the need for improved prevention and early detection. Although stroke is traditionally viewed as a disorder of large-vessels, microvascular dysfunction may strongly influence both the likelihood and consequences of cerebrovascular injury. Because capillaries have extremely narrow lumens and low flow velocities, they are particularly vulnerable to obstruction by leukocytes, rigid or aggregated erythrocytes, platelet aggregates, or fibrin-rich microthrombi. Many pathological states increase the frequency and persistence of capillary stalls, with consequences beyond transient perfusion deficits. Capillaries that repeatedly fail to regain flow may be eliminated through pruning without compensatory regrowth, leading to capillary rarefaction, reduced cerebral reserve, and increased ischemic vulnerability. Thus, capillary stalling may actively contribute to progressive deterioration of the cerebral microvascular network. Conditions that increase stroke risk converge on mechanisms that promote capillary stalling, including endothelial activation, leukocyte adhesion, inflammation, altered erythrocyte rheology, and microthrombus formation. These processes occur across diverse disorders including TBI, AD, diabetes, infection, and cerebral small vessel disease, suggesting that capillary stalling is a shared microvascular pathway linking systemic disease to cerebrovascular injury. Persistent stalling may also worsen stroke outcomes by contributing to no-reflow despite arterial recanalization. Detecting capillary stalling and defining its molecular regulators may reveal new opportunities for stroke prevention and early intervention.

## 1. Introduction

Stroke remains a leading cause of death and disability worldwide, affecting more than 90 million individuals in 2021 (Feigin et al., 2025; Global Health Estimates, 2026). Despite advances in acute treatment, only a small proportion of stroke patients qualify for interventions such as thrombolytic therapy or mechanical thrombectomy, making stroke prevention and early detection crucial for improving patient outcomes. Increasing evidence from human data, animal models, and *in silico* simulation suggests that cerebral microvascular dysfunction plays a central role in both stroke susceptibility and recovery (Erdener et al., 2021; Østergaard et al., 2016; Schmid et al., 2021; Shen et al., 2022; Taylor et al., 2016; Whitehead et al., 2023), highlighting the importance of mechanisms that disrupt blood flow at the level of individual microvessels.

Numerous conditions increase stroke risk by promoting vascular dysfunction. These include common cardiometabolic disorders such as hypertension, hyperlipidemia, diabetes, obesity, sleep disturbance, and congestive heart failure (Martin et al., 2025). Other factors, including traumatic brain injury (TBI), infection, and certain medications, also increase stroke risk through mechanisms that affect coagulation, inflammation, and endothelial function (Albrecht et al., 2015; Barbour et al., 2025; Chen et al., 2011; Eric Nyam et al., 2019; Esterov et al., 2023; Marto et al., 2021; Miller and Elkind, 2016; Turner et al., 2021).

Because the brain relies on continuous blood delivery to sustain its high metabolic demand, even subtle disruptions in vascular function can increase susceptibility to ischemia or hemorrhage (Østergaard et al., 2013). Large vessel pathologies such as atherosclerosis and arterial stenosis contribute to stroke risk by generating emboli, reducing cerebral perfusion, and increasing pulsatile stress on fragile cerebral microvessels (Rovira et al., 2005). Further, growing evidence suggests that microvascular dysfunction may be equally important. Impaired neurovascular coupling and reduced vascular reserve can limit nutrient delivery to neural tissue, contributing to cognitive decline and increasing vulnerability to subsequent perfusion deficits (Vikner et al., 2021). Many stroke risk factors converge on shared microvascular pathways, including endothelial inflammation, leukocyte adhesion, decreased red blood cell (RBC) deformability, and microthrombus formation that impair cerebral perfusion.

One manifestation of microvascular dysfunction that has received increasing attention is capillary stalling, a phenomenon in which transient obstructions disrupt blood flow through individual capillaries (Aspey et al., 1989; del Zoppo et al., 1991; Erdener et al., 2021; Lu et al., 2020; Staehr et al., 2023). Although these events may be brief, their effects can extend beyond the affected vessel. Because capillary networks operate near the limits of oxygen delivery, even small disruptions in perfusion can compromise tissue metabolism and alter local hemodynamics. Capillary stalling has long been recognized as a consequence of ischemic injury (Erdener et al., 2021); its potential role as a contributor to stroke susceptibility has received comparatively little attention.

Capillary stalling has different implications at different stages of disease progression. For instance, stalling immediately following TBI may not contribute to stroke vulnerability as directly as stalling that persists for months after injury, which indicates chronic microvascular dysfunction and potential cerebrovascular injury.

Although capillary stalling is often discussed as a contributor to impaired cerebral perfusion and tissue dysfunction, its precise role within the causal hierarchy of cerebrovascular pathology remains incompletely defined. Many upstream processes that promote stalling, including endothelial activation, inflammation, and altered blood rheology, also independently impair microvascular function, making it difficult to distinguish whether stalling is a primary driver of dysfunction or a downstream manifestation of broader vascular pathology.

At the same time, accumulating evidence suggests that persistent or recurrent stalling may itself exacerbate microvascular dysfunction by disrupting local flow patterns, promoting capillary pruning, and reducing perfusion reserve. Thus, capillary stalling may be best conceptualized as a dynamic intermediate phenotype that both reflects underlying vascular pathology and contributes to its progression. Clarifying these relationships will be critical for determining its mechanistic and translational significance.

In this review, we examine the mechanisms that promote capillary stalling, discuss how diverse stroke risk factors converge on these microvascular pathways, and consider how stalled capillaries may contribute to both stroke susceptibility and poor recovery after ischemic injury (Table 1).

## 2. Capillary stalling

Because capillaries have narrow lumens and low blood flow velocities, they are particularly vulnerable to transient obstruction. This phenomenon was first described in the 1990s with improved resolution in *in vivo* microscopy (Kleinfeld et al., 1998; Villringer et al., 1994). More recently, advances in high-resolution intravital imaging, particularly two-photon microscopy, have enabled visualization of individual capillary flow dynamics *in vivo*, leading to renewed interest in capillary stalling as a contributor to cerebrovascular dysfunction.

Using optical coherence tomography and two-photon microscopy, previous studies have estimated that in the healthy mouse cortex, up to 3% of capillaries may be stalled at any given moment (Erdener et al., 2019; Reeson et al., 2018; Santisakultarm et al., 2014). Although many of these stalls are brief and resolve spontaneously, pathological conditions including TBI, AD, diabetes, infections, and cerebral small vessel disease (cSVD), significantly increase the frequency and duration of these events (Anderle et al., 2025; Sharma et al., 2024).

Capillary stalls arise from a variety of intravascular obstructions. These may include adherent leukocytes, rigid or aggregated RBCs, platelet aggregates, and fibrin-rich microthrombi that physically block the capillary lumen. Endothelial activation and inflammation can further exacerbate this process by increasing the expression of adhesion molecules and promoting interactions between circulating blood cells and the vessel wall (Joussen et al., 2002; Knottnerus et al., 2010). Even small changes in blood rheology or endothelial structure can disrupt normal flow because RBCs are approximately the same diameter as capillaries, relying on their capacity to squeeze through vessels (Lazari et al., 2020; Mohanty et al., 2014).

Even brief interruptions in capillary perfusion can produce localized metabolic stress. The brain is highly vascular; neuronal cell bodies are an average of 15 μm from the nearest microvessel in the mouse cortex (Tsai et al., 2009). Reduced oxygenation in the vicinity of stalled capillaries has been inferred in the murine cortical parenchyma based on polarographic measurements of tissue PO_2_ combined with modeling of oxygen diffusion from capillaries and RBC spacing dynamics (Zhang et al., 2021). Because neuronal metabolism depends on continuous blood flow, stalled vessels must be rapidly recanalized to maintain tissue viability.

Several mechanisms contribute to the resolution of capillary stalls. Hemodynamic forces, including those mediated by changes in local neuronal activity, can wash out obstructions and restore flow (Reeson et al., 2022). Enzymatic degradation, such as fibrinolysis of fibrin-rich clots, can also recanalize blocked vessels (Lam et al., 2010). In addition, immune cells can remove intravascular obstructions through phagocytic mechanisms; endothelial cells may also phagocytose obstructions in a process known as angiophagy (Grutzendler et al., 2014; Lam et al., 2010; van der Wijk et al., 2019).

Importantly, animal models show that capillaries that have previously stalled are more prone to stalling again in subsequent weeks (Cruz Hernández et al., 2019; Reeson et al., 2018; Sharma et al., 2024). These recurrent disruptions in flow may reflect persistent local changes in endothelial function, vascular geometry, or blood rheology. Over time, repeated stalling events may also lead to structural remodeling of the microvascular network, contributing to longer-term alterations in cerebral perfusion.

Although capillary stalling is often described as a localized microvascular event, its consequences can extend beyond the affected vessel. Capillary networks function as highly interconnected flow systems in which pressure and flow dynamics in one segment influence neighboring vessels (Bonney et al., 2024). As a result, obstruction of a single capillary can alter flow distribution across the surrounding microvascular network, potentially reducing perfusion in adjacent capillaries and increasing the likelihood of additional stalls. These network-level effects suggest that capillary stalling can influence flow distribution across the microvascular network, potentially reducing perfusion in adjacent vessels and increasing the likelihood of additional stalling. Repeated stalling events may therefore produce cumulative alterations in microvascular structure and function, ultimately reducing perfusion reserve and increasing susceptibility to ischemic injury.

## 3. Capillary stalling, pruning, and stroke vulnerability

Insufficient blood flow through a capillary can result in its removal from the vascular network through a process termed capillary pruning. When shear stress on a vessel wall drops, endothelial cells retract and migrate intraluminally toward a nearby higher flow branch point where they integrate into the neighboring vessel (Santamaría et al., 2020). If this occurs without compensatory sprouting or vessel regrowth, it causes capillary rarefaction, resulting in a net reduction in capillary density.

Rarefaction occurs naturally during aging, augmenting ischemic vulnerability, but pathological conditions may accelerate or exacerbate this process. The decreased capillary density leaves brain tissue more vulnerable to ischemia, particularly in border-zone regions. Reduced microvascular reserve may therefore amplify tissue injury following vascular occlusion, potentially contributing to larger infarcts and worse functional outcomes.

Notably, vessels may be pruned even after flow is restored, suggesting that transient stalling produces lasting changes in vascular architecture (Reeson et al., 2018). Moreover, because capillary beds function as highly interconnected networks, disruption of flow in a single vessel can alter pressure and flow dynamics in both upstream and downstream vessels, potentially triggering additional stalls (Bonney et al., 2024; Nishimura et al., 2006). As rarefaction progresses and collateral pathways are lost, the remaining vessels become increasingly vulnerable to obstruction. Pathology, including ischemic disorders and neurodegeneration, also alters capillary pruning, promoting hypoperfusion and tissue damage (Santamaría et al., 2020).

Taken together, these observations suggest that capillary stalling may have consequences that extend beyond transient disruptions in local perfusion. Repeated stalling events may gradually remodel the cerebral microvascular network through pruning and rarefaction (Schager and Brown, 2020), reducing perfusion reserve and increasing the likelihood that otherwise modest vascular insults produce tissue injury. In this way, capillary stalling may represent an early microvascular event that links systemic vascular dysfunction to stroke susceptibility. Conditions that promote inflammation, thrombosis, endothelial dysfunction, or impaired blood rheology may therefore increase stroke vulnerability not only by affecting large vessels, but also by progressively destabilizing the capillary network.

## 4. Stroke types

Stroke refers to neuronal injury resulting from disruption of cerebral blood flow and is broadly classified as ischemic or hemorrhagic. Because neurons have minimal energy reserves, the brain depends on continuous blood supply for survival. Ischemic strokes, which account for approximately 80% of cases, occur when vascular obstruction blocks the delivery of oxygen and metabolic substrates to brain tissue. Hemorrhagic strokes result from bleeding within or around the brain. Although large-artery strokes account for much of the mortality and disability associated with stroke, vascular injury can occur throughout the cerebrovascular tree, including within the microvasculature. Dysfunction of small vessels can therefore produce neurologic injury even when major arteries remain patent.

Detecting small-vessel strokes presents a significant challenge. Much of the epidemiological literature relies on administrative datasets, such as ICD billing codes, which typically classify strokes only as ischemic or hemorrhagic and rarely capture detailed clinical information about the size or location of vascular lesions. As a result, many subclinical or “silent” strokes go undetected because they produce minimal acute symptoms and do not prompt medical evaluation (Fanning et al., 2014). Despite their subtle presentation, silent infarcts are clinically important: they may accumulate over time, contribute to cognitive impairment, and increase the risk of future symptomatic strokes (Bokura et al., 2006).

Small vessel pathology also produces distinct stroke subtypes. Lacunar strokes arise from occlusion of small perforating arterioles, producing subcortical lesions that often appear as fluid-filled cavities or “lacunes.” Depending on lesion location, these infarcts may produce subtle neurological deficits or remain clinically silent, and they are often detected only through diffusion-weighted MRI after injury has occurred (Thomas et al., 2025). Transient ischemic attacks (TIAs) represent another manifestation of cerebrovascular dysfunction. TIAs involve a temporary reduction in cerebral blood flow that produces stroke-like symptoms lasting minutes but that resolves without permanent tissue injury. Although transient, TIAs are clinically significant because they indicate a markedly elevated risk of subsequent stroke (Coull et al., 2004; Transient Ischemic Attack (TIA), 2026). Microvascular mechanisms such as capillary stalling may contribute to these transient perfusion deficits by producing localized, reversible disruptions in blood flow that do not reach the threshold for infarction but nevertheless impair tissue function. Recurrent or spatially clustered stalls could also reduce microvascular reserve, lowering the threshold at which subsequent vascular insults produce overt ischemic injury. Together, these observations highlight the importance of microvascular pathology in cerebrovascular disease and suggest that cumulative dysfunction within small vessels may contribute to the development of larger ischemic events (Knottnerus et al., 2010).

Importantly, the relevance of capillary stalling is not limited to small-vessel disease. Following large-vessel occlusion and subsequent recanalization, microvascular perfusion often fails to recover fully—a phenomenon known as “no-reflow” or futile recanalization. Increasing evidence suggests that capillary-level obstructions, including leukocyte adhesion and microthrombi, are key contributors to this impaired reperfusion. In this context, capillary stalling may play a critical role in determining tissue viability and functional recovery even when upstream arterial flow is restored.

Understanding the microvascular mechanisms that disrupt cerebral perfusion is therefore critical for understanding stroke susceptibility. Together, these observations highlight that microvascular mechanisms, including capillary stalling, may contribute to both transient and permanent disruptions in cerebral perfusion across multiple stroke subtypes.

## 5. Capillary stalling in stroke risk factors and associated conditions

Many stroke risk factors and comorbid conditions cause microvascular dysfunction that precedes the development of clinically detectable pathology. Although these conditions differ in their underlying biology, many converge on mechanisms that promote capillary stalling, including inflammation, endothelial activation, altered blood rheology, and microthrombus formation. Oxidative stress can impair RBC deformability and promote adhesion of RBCs to the endothelium, limiting their ability to traverse narrow capillary lumens (Bettiol et al., 2022; Zhang et al., 2023). These oxidatively stressed RBCs may aggregate and obstruct capillaries, potentially leading to microhemorrhages if not effectively cleared, even in the absence of overt blood–brain barrier disruption (Zhang et al., 2023). Importantly, the cellular composition of capillary plugs varies across pathological conditions and may influence recanalization. For instance, a mouse model of type 1 diabetes exhibited capillary stalls caused by obstructions primarily composed of RBCs, while leukocytes caused stalls in healthy control animals (Sharma et al., 2024). Differences in plug composition may therefore influence degradation kinetics, clearance pathways, and endothelial injury, with important implications for the development of targeted therapies.

### 5.1. Traumatic brain injury (TBI)

TBI is an independent risk factor for both ischemic and hemorrhagic strokes, even months or years after injury. However, the mechanisms that contribute to this risk remain incompletely understood. One pathway involves elevated circulating fibrinogen following TBI. Increased fibrinogen promotes vascular permeability and cognitive deficits in experimental models (Muradashvili et al., 2021), and it contributes to thrombus formation by facilitating platelet aggregation (Sulimai and Lominadze, 2020). In addition to its role in coagulation, fibrinogen modulates inflammation by regulating leukocyte behavior and promoting pro-inflammatory cytokine production (Akassoglou and Strickland, 2002). Together, these effects suggest that fibrinogen may promote capillary stalling through both thrombotic and inflammatory mechanisms following TBI.

Experimental data support this hypothesis. In a controlled cortical impact (CCI) model of TBI, platelet-fibrin microthrombi formed in both arterioles and venules of the injury penumbra, impairing local blood flow. Within 120 min of injury, microthrombi were observed in up to 77% of venules and 40% of arterioles (Schwarzmaier et al., 2010). Consistent with these findings, our laboratory also reported an increase in fibrin(ogen) associated with vessel walls in both male and female mice subjected to transient middle cerebral artery occlusion (MCAO) seven days or 28 days after a mild TBI, compared to mice that received an MCAO after a sham injury. The findings suggest that TBI produces lasting vascular dysfunction that increases stroke vulnerability (Whitehead et al., 2023).

TBI also alters leukocyte–endothelial interactions that may contribute to microvascular obstruction. In a mouse model of TBI, leukocyte rolling occurred more frequently in venules compared with sham-injured controls (Schwarzmaier et al., 2010). Large aggregates of leukocytes and platelets were also observed in venules after injury. Although these aggregates formed primarily on the venous side and were therefore unlikely to directly plug capillaries within the injury penumbra, they reflect heightened vascular inflammation that may promote downstream microvascular dysfunction (Schwarzmaier et al., 2010).

Neutrophils also contribute to capillary stalling indirectly through the release of neutrophil extracellular traps (NETs), extracellular chromatin structures that promote coagulation and inflammation. Following CCI in mice, NETs accumulate in regions of hypoperfusion (Vaibhav et al., 2020). In human patients with severe TBI, serum levels of deoxyribonuclease I (DNase I), which degrade NETs, are negatively correlated with intracranial pressure (Vaibhav et al., 2020). NETs also colocalize with extravasated RBCs and necrotic tissue after TBI (Vaibhav et al., 2020) and can promote coagulation through complement activation (de Bont et al., 2019).

TBI additionally produces long-lasting alterations in CBF. Immediately after injury, CBF typically declines due to edema, pericyte dysfunction, and oxidative stress. This reduction is often followed by a transient hyperemic phase; patients who exhibit this increase in CBF tend to have better functional outcomes six months after severe TBI (Inoue et al., 2005). However, chronic reductions in CBF frequently develop and may persist for months or even years after injury. For example, after combat-related brain injuries, veterans showed lower CBF for 3–8 years post-injury compared to uninjured controls (Ponto et al., 2016). Similarly, mice subjected to repeated mild TBI exhibited sustained reductions in CBF lasting at least six months (Lynch et al., 2016). Persistent hypoperfusion, combined with increased microthrombus formation and inflammatory signaling, may create conditions that favor capillary stalling and impaired microvascular reperfusion after TBI.

### 5.2. Alzheimer’s disease and vascular dementia

Stroke and Alzheimer’s disease (AD) share a bidirectional relationship. Stroke increases the risk of developing AD, and individuals with silent infarcts have more than double the risk of developing dementia (Vermeer et al., 2003). Conversely, AD is associated with increased stroke risk, and vascular risk factors such as hypertension and diabetes are especially common in AD patients. Critically, vascular dysfunction is among the earliest detectable abnormalities of AD and often precedes the onset of cognitive impairment (Ali and Bracko, 2022).

AD is associated with impaired CBF autoregulation, vascular structural changes, and chronic neuroinflammation. Many patients also develop cerebral amyloid angiopathy, which contributes to microhemorrhages and microinfarcts (Crumpler et al., 2021). Microinfarcts are particularly common in dementia: 62% of patients with vascular dementia, 43% with AD, and 33% of individuals with both AD and cerebrovascular pathology exhibit microinfarcts, compared with 24% of individuals without dementia (Brundel et al., 2012). Silent infarcts also occur more frequently in patients with dementia, particularly AD (Vermeer et al., 2003).

Increasing evidence suggests that capillary stalling contributes to these vascular abnormalities. Multiple mouse models of AD exhibit increased neutrophil-mediated capillary stalling, and the frequency of stalling does not correlate with amyloid-β burden (Cruz Hernández et al., 2019). Depletion of neutrophils using an anti-Ly6G antibody reduces capillary stalls and improves cognitive performance, demonstrating a causal role for neutrophils in microvascular obstruction in these models. Loss of the endothelial glycocalyx, a proteoglycan layer lining the luminal surface of blood vessels, may further contribute to stalling by promoting leukocyte adhesion to the endothelium (Yoon et al., 2022). In both patients and animal models, reductions in CBF are also commonly observed and may further promote capillary stalling by limiting perfusion reserve. Together, these findings suggest that capillary stalling may represent an important mechanism linking vascular dysfunction in AD to cerebral hypoperfusion and increased susceptibility to ischemic injury.

### 5.3. Diabetes mellitus

Diabetes mellitus (DM) is a major risk factor for stroke and is associated with worse outcomes following ischemic injury (Lau et al., 2019; Mosenzon et al., 2023). Approximately one-third of stroke patients have diabetes, and the disease contributes to cerebrovascular pathology through mechanisms including large-artery atherosclerosis, cSVD–like pathology, endothelial dysfunction, and cardiac embolism. In addition, metabolic disruptions following neurological injury, including TBI, may further exacerbate insulin resistance and vascular dysfunction (Karelina et al., 2016). Some antidiabetic medications, including pioglitazone and glucagon-like peptide-1 receptor agonists, also reduce stroke risk in humans, highlighting the importance of metabolic and vascular interactions in cerebrovascular disease (Mosenzon et al., 2023).

Experimental studies suggest that early microvascular changes in diabetes promote conditions favorable for capillary stalling. In both human patients and animal models, diabetes increases leukocyte adhesion and reduces capillary perfusion. For example, intercellular adhesion molecule-1 (ICAM-1) expression is elevated in the retinal vasculature of diabetic patients and animal models (McLeod et al., 1995; Miyamoto et al., 1999). Similar findings have been reported in animal models, where increased vascular endothelial growth factor (VEGF) expression promotes leukocyte adhesion and disrupts microvascular blood flow. In a rat model of diabetes, VEGF neutralization reduced ICAM-1 expression and decreased leukocyte adhesion in retinal capillaries, arterioles, and venules (Joussen et al., 2002). Inhibition of endothelial nitric oxide synthase (eNOS) similarly reduced leukocyte adhesion in these animals.

Direct evidence for capillary stalling has also emerged from experimental models of diabetes. A recent mouse model of type 1 diabetes exhibited increased capillary stalling compared with healthy controls, with most obstructions composed primarily of RBCs (Sharma et al., 2024). Blocking IL-10 signaling restored blood flow and improved cognitive performance in these animals. In addition, RBCs from patients with type 2 diabetes promote endothelial dysfunction through increased hydrogen peroxide production (Zhou et al., 2018), suggesting that altered RBC physiology may further contribute to microvascular obstruction. Together, these findings indicate that inflammatory signaling, leukocyte adhesion, and altered RBC function may converge to promote capillary stalling in diabetes.

### 5.4. Infections

Physicians have recognized an association between infection and stroke for centuries (Elkind et al., 2020; Macko et al., 1996), but this relationship gained renewed attention during the COVID-19 pandemic. Both acute and chronic infections increase stroke risk through multiple mechanisms, including hypercoagulability, increased leukocyte activation, and endothelial glycocalyx shedding. Clots retrieved from patients with COVID-19 contain higher levels of von Willebrand factor and C-reactive protein compared to clots from non-COVID patients, suggesting endothelial injury and heightened inflammatory activation in addition to coagulation abnormalities (Brinjikji et al., 2023).

Although capillary stalling itself has not been extensively examined in the context of infection, the inflammatory and pro-thrombotic environment associated with infection may promote microvascular obstruction. For example, infections can drive the development of thrombotic microangiopathies (TMAs), which are characterized by microvascular thrombosis and endothelial injury (Merlen et al., 2025). These processes may create conditions that favor capillary stalling by promoting leukocyte adhesion, platelet aggregation, and intravascular obstruction within the microcirculation.

### 5.5. Cerebral small vessel disease (cSVD)

Cerebral small vessel disease contributes substantially to neurodegeneration and accounts for most intracerebral hemorrhages in individuals over 65 and approximately 25% of ischemic strokes (Duering et al., 2023). The disorder is characterized by structural and functional abnormalities of small cerebral vessels, including luminal narrowing caused by collagen and fibrin deposition within vessel walls, chronic hypoperfusion, inflammatory signaling, and blood–brain barrier (BBB) dysfunction. These vascular changes contribute to the development of small, deep subcortical infarcts, lacunes, microbleeds, and enlarged perivascular spaces, which collectively increase stroke susceptibility and ultimately promote brain atrophy (Duering et al., 2023; Dupré et al., 2024).

Experimental models of cSVD provide evidence that microvascular obstruction may precede overt vascular pathology. In spontaneously hypertensive stroke-prone (SHRSP) rats, vessel compression associated with edema after infarction leads to RBC accumulation within capillaries and arteriolar walls, particularly in older females (Schreiber et al., 2013; Schreiber et al., 2012). While control animals exhibit RBC accumulation primarily in capillaries, SHRSP rats display accumulation in both capillaries and arterioles, along with IgG deposition around vessel walls. Notably, these changes occur before the development of other cSVD features such as microbleeds and thrombosis and are observed near constricted smooth muscle cells.

Although MRI can detect many structural features of cSVD, pathology may develop for years before clinical symptoms emerge, often manifesting initially as subtle cognitive decline (Dupré et al., 2024). Early microvascular abnormalities, including impaired perfusion and intravascular cellular accumulation, may therefore represent important contributors to disease progression and may promote capillary stalling within the cerebral microcirculation.

**Fig. 1.**
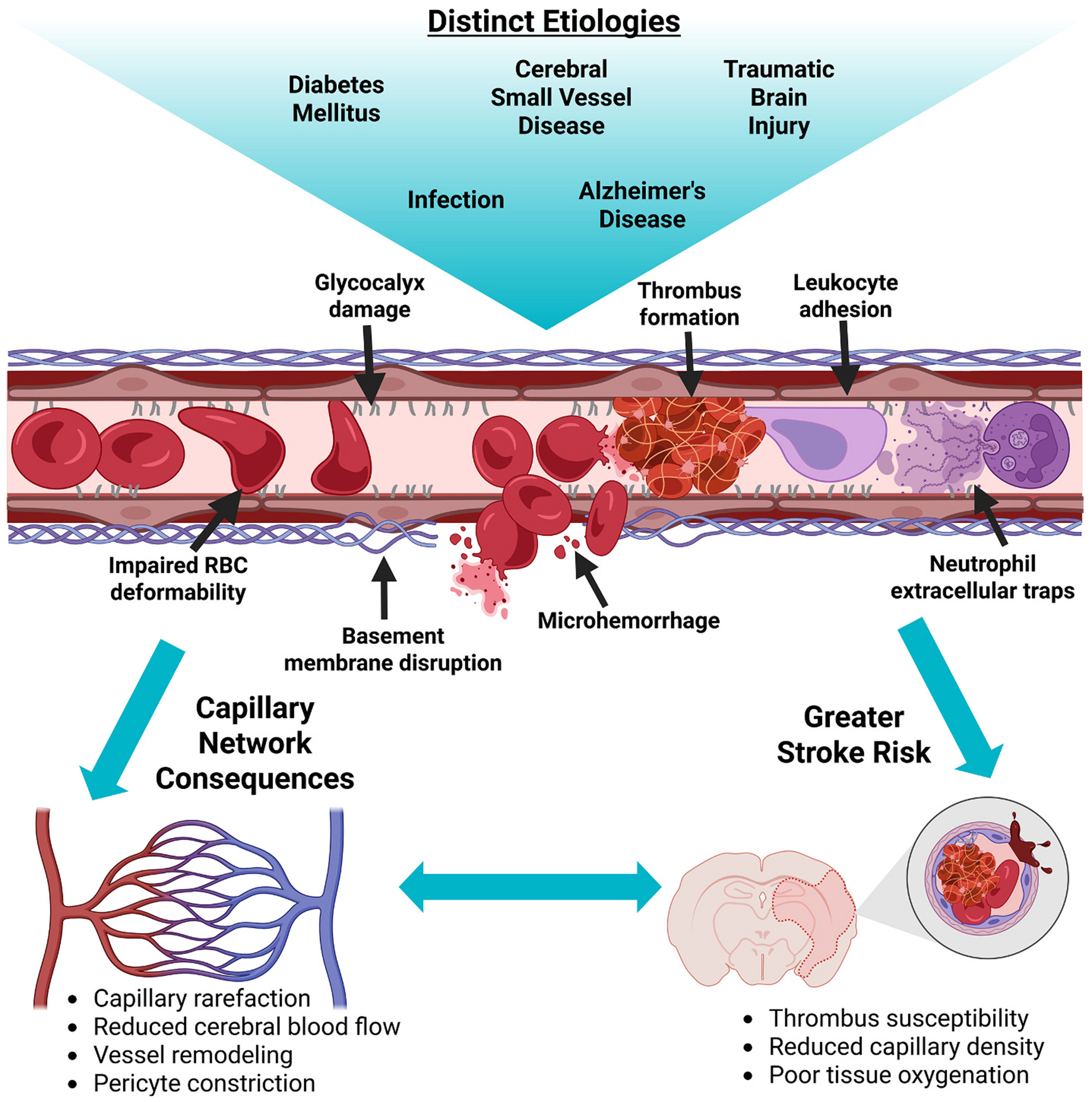
Distinct etiologies share converging mechanisms to promote capillary stalling. Diverse disease states damage the endothelial glycocalyx, promote thrombus formation, impair red blood cell deformability, disrupt the basement membrane, initiate microhemorrhages, reduce cerebral blood flow, promote blood cell adhesion, and alter leukocyte behavior, driving capillary stalling. This disturbs the greater vascular network through dysregulated blood flow and vessel remodeling, resulting in functional and anatomical capillary rarefaction. Capillary stalling also causes poor perfusion and tissue oxygenation. Together, these functional outcomes promote both ischemic and hemorrhagic strokes and exacerbate poor stroke outcomes. (For interpretation of the references to colour in this figure legend, the reader is referred to the web version of this article.)

Despite their diverse etiologies, these conditions converge on several common microvascular mechanisms that promote capillary stalling. Inflammation, endothelial activation, altered blood rheology, and microthrombus formation are recurrent features across TBI, neurodegenerative disease, metabolic disorders, infection, and cSVD. These processes increase leukocyte adhesion, impair RBC deformability, and promote intravascular aggregation, creating conditions that favor transient or persistent capillary obstruction. Because capillary networks operate near the limits of oxygen delivery, even brief interruptions in perfusion may compromise tissue metabolism and reduce cerebrovascular reserve. Capillary stalling may therefore represent a common pathway linking systemic disease and vascular risk factors to impaired microvascular perfusion, increased stroke susceptibility, and poorer recovery following ischemic injury (Fig. 1).

## 6. Capillary stalling and poor stroke outcomes

Following ischemic stroke, the brain undergoes a period of angiogenesis that contributes to neural recovery. However, newly formed microvessels may be particularly susceptible to impaired perfusion. In mice, capillary stalling density doubled in newly formed vessels 28 days after photothrombotic stroke compared with baseline levels (Lu et al., 2020). In addition to disrupting perfusion, capillary stalling can promote capillary pruning and subsequent microvascular rarefaction (Reeson et al., 2018). Loss of capillary density may further reduce cerebrovascular reserve, leaving tissue more vulnerable to future ischemic events and increasing the likelihood of additional stalls.

Capillary stalling also contributes to the “no-reflow” or “futile recanalization” phenomenon, in which microvascular perfusion fails to recover despite successful reopening of an occluded artery (Shi et al., 2014; Staehr et al., 2023). Leukocyte adhesion within capillaries is a major contributor to these events (Amki et al., 2020; Aspey et al., 1989; del Zoppo et al., 1991; Erdener et al., 2021). In a thrombin-induced stroke model, male mice exhibited persistent microvascular hypoperfusion in the penumbra even after clot lysis. Although platelet aggregates and adherent RBCs were occasionally observed, most capillary stalls resulted from neutrophil obstruction (Amki et al., 2020). Depletion of neutrophils using anti-Ly6G antibodies prior to stroke induction significantly reduced capillary stalling, decreased tissue damage, and improved sensorimotor outcomes seven days after injury. Among the remaining stalled vessels, most were obstructed by RBCs. Neutrophils may also worsen stroke outcomes through the release of NETs, which activate microglia, damage endothelial cells, and amplify inflammatory signaling (Ardic and Dinc, 2026).

## 7. Conclusion

Capillary stalling is increasingly recognized as an important contributor to microvascular dysfunction and may represent a key mechanism linking diverse vascular risk factors to impaired cerebral perfusion and stroke susceptibility. However, the heterogeneous mechanisms that drive capillary stalling make it difficult to target therapeutically. Many interventions that reduce stalling in animal models are not readily translatable to clinical practice. For example, neutrophil depletion using anti-Ly6G antibodies reduces capillary stalling and improves outcomes in mice, but human neutrophils do not express Ly6G, making this strategy unsuitable for clinical use (Amki et al., 2020; Erdener et al., 2021). Moreover, neutrophils play essential roles in host defense, and broad suppression of neutrophil function could increase vulnerability to infection, which itself is a risk factor for stroke. Similarly, inhibition of vascular endothelial growth factor A (VEGF-A) signaling reduces capillary stalling in mouse models of AD and microvascular embolism (Ali et al., 2021; Reeson et al., 2018). However, VEGF-A regulates multiple processes including angiogenesis, vascular maintenance, neurogenesis, and neuroprotection, making systemic inhibition an impractical therapeutic strategy.

Identifying capillary stalling in human patients also remains challenging (Table 2). In animal models, *in vivo* microscopy enables direct visualization of cortical capillaries and real-time measurement of blood flow. In humans, optical coherence tomography (OCT) angiography is increasingly used to noninvasively image the retinal microvasculature (Debourdeau et al., 2025; Duan et al., 2022; Greig et al., 2020; Liu et al., 2021), which may provide insight into systemic microvascular health. However, OCT currently has several limitations, including relatively slow acquisition times, variability across imaging platforms, and limited ability to assess additional vascular parameters such as permeability. Improving high-resolution imaging techniques capable of detecting microvascular flow disruptions *in vivo* will be essential for translating experimental findings into clinical practice.

Future work should focus on identifying the molecular and cellular mechanisms that govern capillary stalling across disease states and on developing clinically feasible strategies to detect and modulate these events. In addition, identifying circulating or cerebrospinal fluid biomarkers associated with microvascular obstruction may help identify individuals at elevated risk of stroke and guide therapeutic intervention. Because capillary networks operate near the limits of oxygen delivery, even brief interruptions in flow may have disproportionate effects on tissue viability. A deeper understanding of capillary stalling may therefore reveal new opportunities to improve microvascular perfusion, reduce ischemic vulnerability, and enhance recovery following stroke.

## Figures and Tables

**Table 1:** The table summarizes findings from human and animal studies supporting the hypothesis that diverse disease states promote capillary stalling and microvascular dysfunction that drives stroke risk and poor stroke outcomes.

Study	Population	Microvascular outcome	Followup	Mechanical function	References
Human studies					
Angiophagy	Retrospective review of fluorescein angiographies with retinal artery occlusions	Emboli in the perivascular space next to previously occluded vessels in image series			Grutzendler et al., 2014
Leukocyte-endothelial interactions	Patients with TBI and elevated intracranial pressure (ICP)	Negative correlation between serum deoxyribonuclease-1 and ICP		Neutrophil extracellular traps (NETs) correlate to cerebral edema.	Vaibhav et al., 2020
Chronic CBF reduction	Severe traumatic brain injury	Transient increase in CBF following immediate decrease; chronic reduction			Inoue et al., 2005
	Combat-related traumatic brain injury	Reduced CBF for 3–8 years			Ponto et al., 2016
Animal models				
Capillary stalling	Distal MCAO	Transient RBC and leukocyte stalls during and after ischemia			Erdener et al., 2021
	AD	Decreased CBF		Neutrophil-mediated capillary stalling	Cruz Hernández et al., 2019
	Photothrombotic stroke	Increased number and frequency of stalling		Increased stalling in newly formed capillaries	Lu et al., 2020
	Photothrombotic stroke	Capillary flow stalling		Impaired neurovascular coupling and pericyte contraction	Staehr et al., 2023
Fibrin	Type 1 diabetes	RBC-mediated capillary stalling		Inhibiting IL-10 signaling reduced stalling while stimulating IL-10 signaling increased stalling.	Sharma et al., 2024
	Traumatic brain injury (CCI)	Platelet-fibrin microthrombi		Impaired blood flow in venules and arterioles	Schwarzmaier et al., 2010
	Traumatic brain injury (mild closed head), MCAO	Vessel-associated fibrin(ogen)			Whitehead et al., 2023
Angiophagy	Red blood cell oxidative stress	Microhemorrhage formation and decreased blood velocity for five days after injection of oxidatively stressed RBCs		Erythrophagocytosis	Zhang et al., 2023
	Fibrin clots, cholesterol emboli, polystyrene spheres	Embolic translocation	Reduced with MMP2/9 inhibitor administration	Flow restoration	Lam et al., 2010
	Fibrin clots, cholesterol crystal embolization	Embolic extravasation in the brain, heart, lung, retinal, and kidney microvasculature			Grutzendler et al., 2014 van der Wijk et al., 2019
Leukocyte-endothelial interactions	Traumatic brain injury (CCI)	Leukocyte-platelet aggregates		Impaired blood flow in venules	Schwarzmaier et al., 2010
	Traumatic brain injury (CCI)	Neutrophil extracellular trap (NETs) accumulation		Hypoperfusion	Vaibhav et al., 2020

**Table 2:** The table summarizes techniques used to detect capillary stalling and microvessel obstructions in human patients and animal models along with key findings.

Technique	Model	Imaging Timepoints	Findings	Advantages & Disadvantages	References
Animal Studies					
*In vivo* two-photon microscopy *In vivo* two-photon laser scanning microscopy	APP/PS1 transgenic mouse model of Alzheimer’s disease	Baseline, one hour after anti-VEGF-A treatment, and one week after treatment every other day	Mice treated with anti-VEGF-A showed reduced capillary stall incidence and increased capillary flow speed within an hour of treatment, with effects remaining during treatment every other day for a week.		Ali and Bracko, 2022
	t-PA administration two hours after middle cerebral artery thrombin clot injection	Sixty minutes after t-PA administration and clot resolution	35% of capillaries in the core and 15% of capillaries in the penumbra remain stalled after thrombolysis, primarily caused by neutrophil obstruction, though RBC and platelet aggregates were also present. Capillary stalls reduced reperfusion to only 60% of baseline.	Advantage: clinically relevant t-PA administration	Amki et al., 2020
	Focal capillary irradiation injury	Longitudinal imaging for 21 days	59% of injured vessels regressed back to their branch point 7–14 days post injury; 41% of injured vessels reconnected and reestablished blood flow. Superficial capillaries were more likely to regress. Observed arteriole-capillary transition constriction upstream of regression sites and reduction in blood flow.	Advantages: can compare awake and anesthetized animals; isolated capillary injury without penumbral damage	Bonney et al., 2024
	APP/PS1 and 5xFAD transgenic mouse models of AD		1.8% of capillaries in APP/PS1 mice had stalled flow compared to 0.4% in wild type controls; 5xFAD and TgCRND8 mice show similar elevation. Stalled capillaries were more likely to restall 10× more frequently than predicted. Stalled capillaries showed an outsized impact on cerebral blood flow. Stalling was primarily caused by adherent neutrophils.		Cruz Hernández et al., 2019
	Male Sprague-Dawley rats	128 s recordings	Irregular RBC flow including stalling and occasional direction reversal; RBC flux and speed increase with stimulation. Flow in deeper layers of the cortex is slower.		Kleinfeld et al., 1998
	Photodisrupted single microvessel injuries in adult male Sprague-Dawley rats	Real time monitoring before, during, and after injury	Decreased RBC speed in vessels downstream of intravascular clot	Advantages: images injury and consequences in real time	Nishimura et al., 2006
	Awake and lightly anesthetized young adult and aged male and female *Tek*-GFP mice; young adult male Kdr^fl/fl^ mice; young adult Tek-CreER^T2^ xKdr^+/fl^ mice; 1 μm and 4 μm microsphere injections	Two-hour sessions over 21 days	75–80% of stalled capillaries recanalized within 24 h. 30% of obstructed capillaries were pruned within 21 days, including vessels that previously regained flow. Pruning was especially common in capillaries that stalled repeatedly. 2% of sphere-obstructed capillaries recanalized through angiophagy; most sphere obstructions reentered circulation. Stalling and pruning contribute to reduced capillary density in aging. Capillaries that recanalize have significantly higher or lower expression of VEGFR2 than obstructed capillaries. Stimulating VEGFR2 increases obstruction density. Inhibiting VEGFR2 signaling increases rates of recanalization.		Reeson et al., 2018
	Anesthetized transgenic mouse model of myeloproliferative neoplasms	Repeated imaging at 15, 30, 60, and 120 min	Wild type mice showed stalls in about 3% of capillaries, but increased to up to 27% in myeloproliferative neoplasia models; obstruction composition and recanalization speed differed between models.		Santisakultarm et al., 2014
	Model of type 1 diabetes in male and female mice	2–4 weeks after diabetes induction	Diabetes increases capillary stalling in the brain which is associated with high levels of IL-10 in the serum.		Sharma et al., 2024
	Male C57BL/6 mice after photothrombotic stroke	Prior to occlusion and 0.25, 2, 4, 6, and 8 h post-occlusion.	Penetrating arteriole occlusion generated large infarcts with microvascular impairment that contributed to the expansion of the infarct.		Taylor et al., 2016
	Male Tie2-GFP mice injected with tert-butylhydroperoxide oxidatively stressed or saline-treated RBCs	Imaging 1–4 h, 24 h, 5 days, and 7 days after injection.	Oxidatively stressed RBCs stall more than saline-treated RBCs and trigger microglial reactions, promoting the development of microhemorrhages.		Zhang et al., 2023
*In vivo* two-photon and fixed tissue microscopy (transmission electron microscopy and confocal)	Young adult and aged (22 months) mice injected with microemboli injections of fibrin clots, cholesterol emboli (8-20 μm diameter), or polystyrene microspheres (10-15 μm diameter)	Two, three, five, and eight days after embolization	Microemboli that fail to be washed out translocate outside the vessel in 2–7 days, reestablishing blood flow; rate of extravasation is decreased when inhibiting matrix metalloproteinase 2/9; aged mice extravasate slower.	Advantages: TEM confirmed endothelial projections covering emboli.	Lam et al., 2010
*In vivo* two-photon microscopy, optical coherence tomography (OCT)	Transient distal MCAO C57BL/6 mice	OCT at baseline, ischemia, and 1 h, 2 h, and 24 h reperfusion; two-photon two hours after reperfusion	Reduced capillary perfusion in the penumbra one hour after recanalization (OCT); two photon identified leukocytes.	Advantage: OCT images several hundred capillaries simultaneously.	Erdener et al., 2021
	Male mice modeling subcortical vascular dementia using asymmetric carotid artery stenosis		Approximately 38.4% of vessels stalled repeatedly during 9-week imaging period. Stalled capillaries showed reduced endothelial glycocalyx. Stalling increased when glycocalyx was enzymatically degraded. Stalling primarily due to leukocyte plugging.		Yoon et al., 2022
OCT	Female young adult CD1 and C57BL/6 mice	Nine-minute recordings; imaged after acute cranial window, chronic window in awake animals, chronic window in awake animals and after anesthesia	~0.45% of capillaries stalled with average duration ~15 s, but could last up to a minute. Acute window preparations and isoflurane increased number of stalls. Stalled vessels stalled again a month later.	Advantage: OCT is faster and has wider field of view than two-photon and does not require a contrast agent. Compares awake and anesthetized mice.	Erdener et al., 2019
	Male C57BL/6 mice with photothrombotic stroke	Longitudinal imaging of awake, resting mice over 28 days; one day before and three, eight, 14, and 28 days after photothrombosis	Capillary network reorganization and increased stalling surrounding the ischemic lesion up to four weeks after injury	Advantage: OCT can be used to image damaged BBB because of no need for fluorescent dye.	Lu et al., 2020
Single Photon Emission Computed Tomography (SPECT), MRI	Male spontaneously hypertensive stroke-prone (SHRSP) rats and Wistar rat controls	MRI every second week from 12 to 42 weeks of age; SPECT when abnormalities were detected with MRI	RBCs accumulate in capillaries and arterioles preceding the development of other vascular pathology as early as 12 weeks of age in SHRSP rats.		Schreiber et al., 2012
Post-mortem pathology	Middle cerebral artery occlusion in adolescent male baboons	Tissue collected after one hour of reperfusion following three-hour occlusion	Polymorphonuclear leukocytes present in the microvasculature following ischemia and reperfusion	Disadvantages: post-mortem, one timepoint	del Zoppo et al., 1991
Intravital Microscopy	Male C57BL/6 mice with controlled cortical impact (CCI) TBI	Baseline imaging before CCI, then 30, 60, 90, and 120 min after TBI	Leukocytes and leukocyte-platelet aggregates in venules immediately after TBI; microthrombi in venules and arterioles 30 min after TBI, reducing blood velocity.		Schwarzmaier et al., 2010

Technique	Patient Population Imagin	Timepoints	Findings	References
Human Studies				
Transcranial Doppler Monitoring	Patients with suspected acute ischemic stroke	One hour bilateral, stationary monitoring, followed by two hour unilateral ambulatory monitoring, beginning within 24 h of symptom onset; unilateral monitoring repeated 18–36 h, 48–72 h, and 3 months after symptom onset.	Microemboli detected in 60% of patients	Aarli et al., 2024
[15O] Water Imaging	21 TBI-injured or healthy control male combat veterans, 3–8 years after injury		After TBI, veterans had lower global cerebral blood flow compared to the non-TBI group.	Ponto et al., 2016
CT perfusion	Patients with DWI confirmed lacunar stroke		Perfusion abnormalities were identified in 58% of cases when reviewed blind to DWI, though this increased to 78% when reviewed in conjunction with DWI.	Thomas et al., 2025
Retinal OCT angiography	Stroke patients and age-matched controls	1–419 days after first stroke (median 13 days) and 1–183 days after last stroke (median 11 days)	Decreased retinal vascular density in stroke patients	Liu et al., 2021
	Patients diagnosed with lacunar stroke, nonlacunar infarction, and healthy controls		Lower vascular orientation distribution in superficial capillary plexus is associated with patients with a lacunar infarction compared to nonlacunar infarction. Stroke patients had irregular foveal avascular zones.	Duan et al., 2022
	Acute ischemic stroke patients and age-matched and sex-matched controls	Day 1-day 7 after stroke symptom onset	Stroke patients showed lower parafoveal superficial capillary plexus vascular density and flow index compared to control patients.	Debourdeau et al., 2025

## Data Availability

No data was used for the research described in the article.
